# Negotiating cancer preventative health behaviours and adapting to motherhood: the role of technology in supporting positive health behaviours

**DOI:** 10.1080/17482631.2020.1811533

**Published:** 2020-09-18

**Authors:** Caitlin Notley, Emma Ward, Angelos P. Kassianos, Allison Kurti, Fiona Muirhead, Dian Nostikasari, Jamie Payton, Claire Adams Spears

**Affiliations:** a Addiction Research Group, Norwich Medical School, University of East Anglia, Norwich, UK; b Department of Applied Health Research, UCL, London, UK; c Department of Psychiatry and Psychological Science, University of Vermont, Burlington, VT, USA; d Psychological Sciences and Health, University of Strathclyde, Glasgow, UK; e Kinder Institute for Urban Research, Rice University, Houston, TX, USA; f Department of Computer and Information Sciences, Temple University, Philadelphia, PA, USA; g Department of Health Policy and Behavioral Sciences, Georgia State University School of Public Health, Atlanta, GA, USA

**Keywords:** Cancer prevention, health inequalities, motherhood, identity, technological intervention

## Abstract

**Purpose:**

Across the UK and USA, postpartum smoking relapse rates are high, and rates of breastfeeding and physical activity are low. This project aimed to explore these interrelated health behaviours and technology use, for intervention development to support postpartum cancer prevention.

**Methods:**

Focus groups and interviews with 26 purposively selected women (15 in Vermont, USA and 11 in Norfolk, UK). Recruitment was from deprived areas experiencing multiple disadvantage. Qualitative data were thematically analysed from dual cultural perspectives, underpinned by the social ecological model.

**Results:**

Women negotiate interrelated lifestyle behaviours as part of managing an identity in transition, moving through stages of disturbance, adaptation, acceptance and integration towards “becoming” a new Mother. Technology was integral to women’s process of engagement with mothering identities. Intersectionality underpins complex patterns of interrelated behaviour.

**Conclusions:**

There is scope to improve electronic/digital support for postpartum women cross-nationally to promote interrelated cancer-preventative lifestyle behaviours.

**Abbreviations**

CDC: Center for Disease Control, US; PA: Physical activity; SES: Socioeconomic status; SVI: Social Vulnerability Index; UK: UK; US: USA; WIC: Women infants and children office.

## Background

Pregnancy can motivate positive health behaviours, including smoking cessation. Nonetheless, while women might wish to remain abstinent, up to 75% of ex-smokers return to smoking within 12 months of childbirth (Orton et al., 2017). Reducing postpartum smoking relapse would significantly improve both women’s and children’s health and make a positive impact on cancer prevention efforts. Maternal smoking is the primary cause of infant and child second-hand smoke exposure, which is directly linked to cancer risk (Kim et al., 2018). Maternal smoking also increases the likelihood of children becoming smokers themselves later in life, representing generational transmission of cancer-causing lifestyle behaviours (Gilman et al., 2009).

Evidence suggests interaction between smoking and breastfeeding. Maternal smoking is associated with shorter breastfeeding duration (Thulier & Mercer, 2009), and not initiating or intending to breastfeed is an independent predictor of smoking relapse postpartum (Rockhill et al., 2016; Simmons et al., 2014). Independently of smoking status, breastfeeding is associated with numerous cancer prevention benefits, including reduced risk of maternal breast and ovarian cancers (Chowdhury et al., 2015). However, in the UK, only 1% of women are exclusively breastfeeding at 6 months (Breastfeeding in the UK, UNICEF, 2019).

Additionally, physical activity (PA) has been associated with reduced cigarette craving and lower likelihood of smoking relapse (Roberts et al., 2012). Pregnancy places women at high risk for insufficient physical activity (Downs & Hausenblas, 2004), and approximately two-thirds (64%) of postpartum women do not meet recommended guidelines for daily PA (Albright & Nigg, 2005). The multiple health benefits of physical activity (PA) are well-established, with evidence showing that it can prevent the development of over 30 diseases and conditions, including colon, breast, and endometrial cancers (Booth et al., 2012; Warburton et al., 2006). As such, inactivity is an important modifiable cancer risk factor (Lee et al., 2012).

Willingness and ability to engage in PA and breastfeeding are affected by individual level, physical, and sociocultural level determinants in the larger community environment (Belon et al., 2014). Lack of physical space and facilities can also be barriers to PA (Bauman et al., 2002) and breastfeeding (Downs & Hausenblas, 2004). Reducing both physical and psychological barriers is necessary to promote more active lifestyles (Businelle et al., 2013; Curtin & Matthews, 2016). Overcoming psychological barriers in managing healthy lifestyles includes having emotional, informational, and instrumental social support et al. 2019; Thornthon et al., 2006.

The interlinked cancer risk behaviours of smoking relapse, limited breastfeeding, and physical inactivity are disproportionately represented among those of lower socioeconomic status (SES), thereby contributing to health inequity. Lower income and educational attainment are strongly associated with higher rates of smoking during pregnancy, and relapse to smoking after childbirth (Delpisheh et al., 2006; Higgins et al., 2009). Low SES has also been linked to lower rates and shorter duration of breastfeeding (Flacking et al., 2007; Heck et al., 2006). Additionally, low-SES postpartum women are less likely to meet recommended guidelines for PA (Wilkinson et al., 2004).

Pregnant and postpartum women are high-level users of new technologies to support health behaviour change (Belon et al., 2014). Engagement with technology might include wearable activity trackers, or apps that track baby feeding patterns to support breastfeeding. The proliferation of technology has offered postpartum women widening opportunities to seek help and support as they adjust to motherhood. Becoming a mother is a unique health-related experience, and online communities, or “intimate mothering publics” serve as an important resource for women looking to share experiences or test out new mothering identities in a virtual social space (Johnson, 2015). Social media can provide validation and “invisible” support where it is needed (*Taking the Village Online, 2016*), although can also perpetuate unrealistic discourses of motherhood (Evenson et al., 2009), putting women under significant, socially influenced, pressure.

There is clear need to support postpartum women to engage in interlinked cancer preventative behaviours, with potential to impact both maternal and child long-term health outcomes through cancer prevention. Digital and electronic means of support offer great potential to deliver targeted, tailored support to individuals within their unique social and cultural contexts. This qualitative study sought to understand women’s experiences of health behaviours postpartum, their interactions with technology and electronic support, and their thoughts about new ways in which technology might promote cancer prevention.

## Methods

### Design

Exploratory qualitative study taking a combined deductive and inductive analytic approach.

### Participants

The study recruited postpartum women across UK and US pilot sites (Norfolk, UK and Burlington, Vermont, USA). Sites were chosen as comparable populations with geographically similar semi-rural communities, offering cultural and context-specific comparison. Participants were women from low-SES areas where our team had established connections, recruited via Children’s Centres in the UK and a “women infants and children” (WIC) office in the US (appendix A). We collected minimal demographic data from participants to increase the likelihood of women agreeing to participate in the project, minimizing stigma and maximizing anonymity.

All women recruited were recently postpartum, with babies less than 24 months old. Indices used to measure social vulnerability of the recruitment areas showed that the UK women were in the top 10% of deprived small areas (English indices of deprivation 2015), and the US women were from areas with moderate to high levels of vulnerability (2016) (appendix A). Women we spoke to were 14 white Americans, 10 white British women, one Asian/white American, and one white European. Across the US and UK sites, eleven women in total were former smokers (quit for pregnancy), ten were never smokers, and five were current smokers.

### Data collection

Four focus groups (two UK and two US; n = 22 total focus group participants) and eight individual interviews (three UK and five US; n = 8 interview participants) were conducted with postpartum women. Focus groups included a minimum of three women and lasted up to 120 minutes. Participants were reimbursed for travel costs. The concept of *saturation* determined when to finalize data collection, as a judgement by our research team of the point at which no new views were emerging from data collection.

Focus group topic guides were developed based on the social ecological model (Bronfenbrenner, 1979), addressing barriers and facilitators related to smoking abstinence, breastfeeding, and physical activity (at the intrapersonal, interpersonal, organizational, community, and societal levels); perceived connections between the three cancer risk behaviours; knowledge of existing digital and electronic resources; interest in intervention methods; alternative resources/tools; and suggestions for promoting sustainability. Although our interview guides afforded women the opportunity to discuss cancer prevention lifestyle factors as separate behaviours, in reality these behaviours were inextricably linked. This suggests an interpretation of the situated reality of postpartum women’s cancer preventative health behaviours that embraces complexity, interrelationship, culture and context, including the context of virtual and online social spaces that validate, support and influence behaviour.

### Data analysis

Focus groups were audio-recorded and transcribed verbatim. Transcripts were managed and coded using QSR International’s NVivo software (v11). Data coding and analysis were undertaken by UK and US researchers, independently, following both inductive and deductive approaches (Ayres et al., 2003; Ryan & Bernard, 2003). Initially, codes were developed from the interview topics and the conceptual framework according to layers of influence outlined in the social ecological model. Additional inductive thematic codes were identified from concepts emerging from the data, following a thematic coding approach (Braun & Clarke, 2006). Four coders (two UK and two US) independently coded the transcripts and then met to discuss findings, resolve inconsistencies, and develop the preliminary coding scheme. The remaining transcripts were independently coded, with double coding of all data undertaken independently. Differences between US and UK groups were noted.

Ethical considerations: Ethical approval for the study was granted through the Faculty of Medicine and Health research ethics committee at the University of East Anglia (Reference: 2017/18–12), and equivalent IRB approval in the USA (reference: UVM CHRMS 17–0673), conforming to the ethical principles for medical research on human beings set out in the declaration of Helsinki (World Medical Association, 2013). All participants received full information about the study prior to giving informed consent and were given the opportunity to discuss the study with a researcher and have any questions answered. It was emphasized that participants were free to withdraw from the study at any time, without giving a reason. All identifiable data were treated as strictly confidential and destroyed following the study. Anonymized data only were shared amongst the research team for analysis.

## Results

For the participants, becoming a Mother was experienced as a shift in identity, away from an independent sense of self, towards integrating a new relational self, with their infant, but also in relation to partners and social support networks, both “real” and “virtual”, through the use of technology. Analysis revealed four core “transitional moments” (defined as periods of change and adaptation, discreetly identifiable but gradually unfolding across the process of adaptation to Motherhood), for women in both the UK and USA (Figure 1). In each of these transitional moments, technology was used throughout the process of identity change. Findings are reported below according to these transitional moments, which are fluid and, despite similarities, individually experienced by women within their unique contexts.

**Figure 1.**
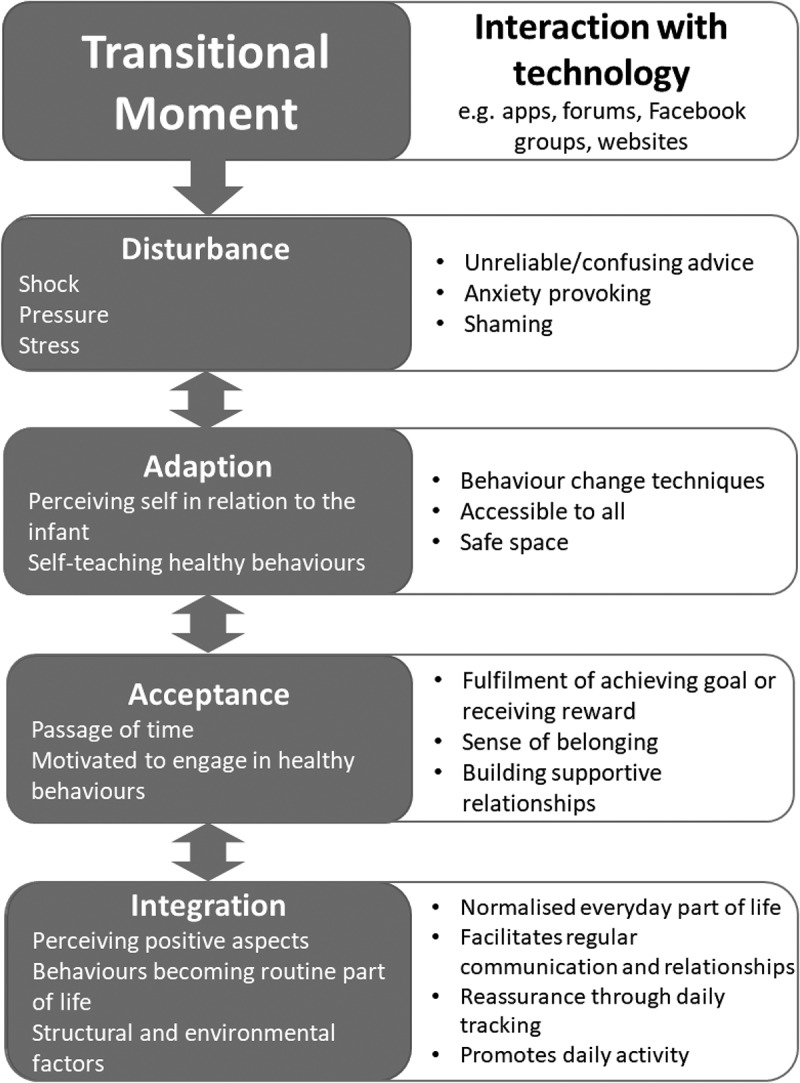
Thematic structure displaying transitional moments relating to cancer preventative health behaviours, including how technology interacts with each moment.

### Disturbance

#### Shock

For study participants, pregnancy and the immediate postpartum period were experienced as a shock, disturbance or disruption to life as they knew it. Disturbance was experienced as traumatic by some due to being completely unprepared for the reality of becoming a mother. There was an expressed sense of shock and disbelief:

“No-one tells you it’s hard”. (US FG1)

### Pressure

For the first time ever, decisions about health behaviour were forced upon them. There was pressure to consider the health of another, the foetus or young infant:

“I went to stop smoking meetings but that felt forced on me as well. It felt like ‘you have to stop smoking right now’ and obviously they went in to how bad it was for the baby and that, but then he’s here, and he’s fine. It made me feel guilty, but the stress of being pregnant and his father wasn’t around, so I was all on my own, so I didn’t stop smoking.” (UK FG 2)

### Isolation

Here the participant draws attention to the pressure from others to change her behaviour by quitting smoking, but also the difficulty of achieving this in isolation, without support. This was reiterated by others in relation to postpartum relapse prevention support:

“I’ve not had anything [any support for staying quit postpartum], I’ve not been given anything in the sense of, like post having a baby, wanting to go back to smoking or anything. Nothing’s been mentioned.” (UK Interview 1)

Similarly, in the US, smoking cessation support postpartum was minimal and impersonal:

“They do give you that little paper with the 1-800 quit smoking number on it. I’ve never called it.” (US interview 3)

### Stigma

Disturbance, in the form of shock or trauma, was experienced by all to a greater or lesser degree following childbirth. This was difficult for women to discuss. Experiences felt stigmatizing and “swept under the rug”, but were an impediment to engaging in healthy lifestyle behaviours for at least some women:

“but … the other thing that’s really making … that makes it hard for me is like childbirth injuries. Like I’m just not better yet and I don’t know, you know, I don’t know if I’m an outlier in that or not, because people like don’t really talk about it.” (US FG2)

The disturbance to identity in becoming a new Mother, combined with the efforts that may have been made to actively engage in health behaviours during pregnancy, made it extremely difficult for women to maintain positive behaviour change postpartum, particularly when the “disturbed” sense of identity did not fit with the trajectory of women’s lives in terms of the behaviour, culture, and expectations of her immediate family and social group:

“So then I stopped [smoking] again, and then we came home for Christmas, and all my family smokes, so I started picking it up a little bit again. It wasn’t helpful that [name of partner] wasn’t like … I mean it’s not that he wasn’t supporting me not smoking, but he was still going behind my back and smoking even though he said he would quit with me.” (US interview 2)

Adjustment to the mothering identity was helped or hindered by infant behaviours, in terms of temperament and feeding patterns, for example. This suggests that the process of transitioning to a new mothering identity is relational, universally experienced, transcending cultural differences, where the needs of Mothers and infants clash, correspond, or move between two extremes:

“I’m the one with the screamer. I try to avoid … he’s pretty good today because he’s so tired but, you know, guaranteed he is the one that would just scream non-stop. And to be honest, kind of exercise is the only break I get from the kids, so I kind of maybe wouldn’t want to do it with them. I know that sounds awful. I mean we mess around at home and do like little YouTube things with my eldest but for a proper, you know, a proper sort of kind of exercise and I just kind of … I want to do it on my own. I want to stick my headphones in and kind of go for it.” (UK FG1)

For many, adjusting to breastfeeding, being a “nursing mother”, was particularly hard and experienced as disturbance to identity:

“I hated pumping in my own house. One time I was like … my husband was like trying to help me out and he was trying to come in keep me company and I was like ‘get out, I’m so embarrassed, I hate doing this’ like, I don’t even want anyone to see me” (US FG1)

### Culture

A culture of “pumping” as part of breastfeeding was something noted particularly in our US sample. It was described how the workplace was set up to support continued breastfeeding:

“So we have a lactation room in my office and so there’s … I think there’s 3 of us currently that are working there that are breastfeeding so we just rotate who is using the room. We have like a college dorm mini-fridge in the room and it’s actually decorated with like antique milk bottles and stuff, it’s kind of funny. Yeah, so it’s just a chill space and there’s a speaker if you want to like plug music in or whatever” (US FG2)

This was entirely different to the UK, where our participants hardly discussed expressing milk. In the UK data, there were more discussions of formula feeding, and a sense overall of less support to breastfeed:

“I did feel quite let down by my midwife … because every time I was mentioning I was struggling she was more suggestive towards formula than she was to carrying on with breastfeeding. Which was a shame because I really wanted it to work” (UK FG1)

### Discourse

Societal discourses of what constitutes a “good mother” impacted self-esteem and influenced difficulty adopting the mothering identity. Discourses were similar across UK and US settings:

“part of the reason why I’ve just quit all of a sudden, because I was so sick of the negative vibes that were associated with breastfeeding, that I just decided to go all in. Because it was really beating me up emotionally because I wanted to breastfeed her, and it was my intention, but my body didn’t agree with me and there wasn’t enough of that other side of the coin saying ‘it’s ok, there’s other people like you, and you just have to do what’s best for you,’ and I wanted to adhere to that idea that ‘breast is best’, and ‘this is what my baby needs’, and ‘my body can do this’, and it’s not always true. So I think that I definitely love how in our culture it’s so supportive in the way it is because I do think it’s something I’d like to do next time if I can but I also feel like there’s such a stigma with not doing it because of that, and it’s kind of unfair. I think a lot of my feelings that I had towards myself that were negative were because of this, like you said, because of this impression that I wasn’t good enough, because I couldn’t do this for my child”. (US FG 2)

### Stress

The postnatal stress many of the mothers experienced linked to disturbance to their identity and influenced their health-related behaviours. They could rationalize the benefits of not smoking, breastfeeding and physical activity, but for some, their current emotional needs meant that they did not feel they had the mental strength to participate in them:

“My husband was very educated certainly about the benefits of exercise. Also about the benefits of breastfeeding too. But I think like emotions overrode all of that, so like for instance, they send like formula samples in the mail, so he was like ‘oh we should keep those just in case’ and I was like ‘no I don’t want to keep them’ and he was like ‘oh but … ’ because he was worried about my level stress, right, so he was like ‘we should keep them, just in case, what if you really need it” (US FG 1)

### Technology

Given the disruption to identity, compounded by a reported lack of support, women often turned to technology for reassurance or validation in navigating new motherhood roles and guiding health decisions. Technology may be the only way to access support, due to time and mobility limitations of being a new mother. In this way, technology such as access to postpartum online message boards or YouTube videos can be considered instrumental in shaping health behaviours. Unfortunately, technology could also sometimes provoke anxiety, hindering transition to confident motherhood identity:

*“I was having trouble getting a latch with little’un, I really struggled to find help if I couldn’t get hold of someone from Medicom to show me how to latch. And I went on to YouTube and I went to like the NHS website to show to get good latches, but it always seemed easier on a video than it was in real life. So that instantly put me off and that was quite frustrating”*. (UK Interview 1)

The technology engaged with the most across both US and UK samples were Facebook groups and online forums (e.g., mumsnet, The Bump). Some women felt the relative anonymity of groups allowed people to postjudgemental attitudes that may not have been expressed face-to-face. This was experienced as “shaming”, perpetuating discourses of good/bad mothering, potentially impacting self-esteem, possibly contributing to further isolation, and hindering adopting a positive motherhood identity:

*“It was [forum] and then I was like, ‘this place is terrible’ … I’m sure there’s all these topics on there, you might find like a nugget of something that’s useful interspersed with like people’s … Yeah, people saying mean things”*. (US FG 1)

Groups and forums sometimes offered advice that was not based on evidence. This was confusing and undermined confidence in decision-making:

*“Advice can be self-serving sometimes and it can also be inaccurate and that’s where it gets scary. You know, somebody’s own opinion if, you know, on low milk supply if they say “oh well eat a jar of Vaseline that’ll help you”, you know, it’s not going to hurt them but it’s not going to help them. You know, like everyone has their own like folk knowledge on things”* (US Interview 5)

Women commented that they would feel more reassured by the advice given if they knew the groups and forums were moderated by health care professionals:

*“I think it would definitely help to have somebody that knows what they are talking about just in case unsafe advice is given”*. (US FG 2)

### Adaptation

As women adapted to the reality of a changing identity whilst transitioning through the postpartum period, adaptation to the disturbance seemed to occur in their narratives. Technology was discussed in a way that mothers felt could help them adapt to a new motherhood identity whilst simultaneously supporting healthy behaviours. The technology employed a range of different behavioural change techniques to support self-teaching of healthy behaviours. These techniques included goal setting and feedback (e.g., pedometers, stop smoking apps); social support (e.g., meet-up apps, Facebook groups, forums); and instructions on how to perform healthy behaviours (e.g., breastfeeding videos, websites on safe postnatal exercise).

### Limited autonomy

Technology to support healthy behaviours in the postnatal period was easily accessible compared to other types of support, which was particularly useful when personal autonomy in the new motherhood role was limited:

*“I think we’re lucky like in the day and age that we live in because a lot of things are done online, like with apps and things like that. And I think you can have all the help and support you need just in the palm of your hand”*. (UK FG 1)

Accessibility was seen as especially important for breastfeeding mothers who often needed help out of office hours:

*“You know when you’re having issues with breastfeeding it’s scary, it’s a lot of the times dark, it’s the middle of the night and you’re freaking out because you haven’t slept in four or five days. And it can be scary … I don’t know what moms did before smartphones because you just look stuff up, you watch YouTube, you do whatever, you’ve got a baby stuck to you for like twelve or twenty-four hours. And I think that for new moms that were struggling that would probably be helpful because … a lot of people don’t call their health care professionals during the night”*. (US interview 5)

One woman felt that the anonymity technology offered could be beneficial in helping mothers adapt to their new role without fear of being judged:

*“I have some younger friends who are first time moms and they’re really anxious to talk to their doctors because of … just their …. like one girl she’s like eighteen, and so she feels very stereotyped, so she would come to me to talk about her issues as opposed to talking to her doctor. But I think that if she had an app like that she’d probably be even more inclined to ask for the help of strangers behind a screen”*. (US interview 5)

### Shifting motivation

Adaptation also occurred through shifting motivation and self-teaching of new healthy lifestyle mantras. These were focused less on the self in isolation and more on the self in relation to the infant:

“My main motivation was her and I eventually had to just to tell myself like literally, it sounds crazy, but I just convinced myself that there were no cigarettes left in the world.” (US interview 2)

Adaptations were intersectional. Here, a participant discusses the complexities between negotiating breastfeeding and the needs of the infant, with her own need to engage in physical activity:

“So I actually … I think breastfeeding makes it hard because I have to take him everywhere, he’s very attached to me. Maybe if … it’s just my support team isn’t really strong. So I just am basically with my baby. So I put him in a stroller and I walk … So I’ve been more active lately than I usually have been”. (US, interview 3)

But complexities linking postpartum health behaviours also made adaptation difficult:

“I think the breastfeeding and the activeness bit is probably more of a connection than the smoking for me, because when I was breastfeeding, like I say, she was actually on for up to three hours at a time, so I just sat there feeding her”. (UK FG 1)

### Environment

The physical built environment could constrain health behaviour. This was especially apparent in the US dataset:

“I moved back from [name of place] and [name of place] they don’t have sidewalks where I was living, so walking down the street with your baby is not an option. So I drove everywhere and so, being back here, I’m able to walk more places.” (US Int 3)

Interpersonal level social factors, in terms of dominant opinions expressed within social groups, including online and virtual peer groups, were influencers on women’s ability to adapt to the new mothering role:

“The opinions coming from the moms stressed me out … I just want them to love him and know that I’m doing the best that I can, and keep your opinions to yourself.” (US FG1)

In this sense, just as negative reactions hindered adaptation, positive and supportive social influence aided the process of adaptation towards acceptance of the mothering role.

### Acceptance

The broad theme of acceptance included beginning to learn what works and does not work for increasing motivation and health behaviour change in the context of life as a mother. For the women in our sample who had managed to quit smoking, there was acceptance that eventually, at the right time, motivation to quit had driven behaviour change:

“The second time I didn’t [use the stop smoking service] and I didn’t need it either, I was just in the right mind-set.” (UK FG 1)

### Time passing

Simply, the passing of time facilitated increasing acceptance of the mothering role. This was aided by the baby’s development, such that mother and baby, in relationship, began to find and accept a new norm:

“We were getting up every 2 hours to nurse all night for months and months and months. And that has stopped so that’s … we’re enjoying it a lot more now”. (US FG1)

### Technology

Technology could also help mothers to accept new healthy behaviours, for example, through personal fulfilment of achieving a goal or receiving a reward. Pedometers were used by many of the women, who enjoyed monitoring their steps and receiving feedback about their activity levels:

“I look at it and I’m like ‘wow, I’ve done four thousand steps’ and I’m like ‘how the hell did I manage to do that. I didn’t even do nothing today’. But then you look back and you’re like ‘actually I may have actually done that many steps’ … because obviously like [name of child] just gets everything out. So I clean the living room and then it’s all out again within like twenty seconds … and I look at my steps and I’m like ‘I did quite a lot thank you’ [laughs]. (UK FG 2)

Women in our sample were motivated by setting achievable walking goals that could be incorporated into their daily routines with their child. This theme was similar across the US and UK datasets:

“I think that those are very helpful things because it gives you a way to track yourself and to monitor yourself. And like, I know when I start like training for a run or something, since I always have my phone on me, a lot of times I’ll do like when I’m running errands or something I’ll just hit my run app for walking and then just see how far I’ve gone while running errands, or just while walking throughout the day … having something that measures your progress in real time is definitely helpful I think”.(US interview 5)

### Renewed motivation

Rewards relating to their new motherhood role also influenced acceptance of healthy behaviours. Women noted that their motivation for quitting smoking might be reinforced via an app that showed how much money they had saved by not spending money on tobacco. They felt it would be especially motivating if the app visually represented saving towards something for their family:

“I mean, if it was for something for the family in the sense of like a day out for the kids, taking them somewhere like [family attraction], you know, money off towards that, that would be awesome because then everyone could benefit from that. That wouldn’t be just me. It’s great that it could potentially just be mum but I would look at something that would probably benefit the family as an entity rather than just me”. (UK Focus Group 2)

### Relationship building

A further way in which technology promoted acceptance of healthy behaviours within the mothering role was by providing women with a platform for building relationships. It gave them an opportunity to find their “mom tribe” and develop a sense of belonging, cementing motherhood identity. Although, as described above, there were some reported negative experiences of Facebook groups and forums, these could also be an accepting and supportive environment in which to seek advice, but also to reach out to other mothers, reducing feelings of isolation:

“I’ve got Facebook on there for like social networking. There’s a couple of groups on there, it’s called ‘exclusive pumping mummies’. They’re based in the US and it’s a support group for mums who have tried breastfeeding, have had trouble and now express off. We can all go there and vent and talk about our day” (UK Interview 1)

Generally, digital social support may impact the felt sense of support, and therefore wellbeing, which in turn may facilitate and underpin engagement in healthy behaviours. One woman had used groups not only to connect with other mums virtually, but also to meet up in the “real world”:

“That’s just like the Facebook group that I’m in. Because they’re like … they’re from all around the country and some are like in America and that and some come from abroad. But yeah they ask if anyone’s local and would like to meet up for a coffee and then you get six or seven people who reply to you and say ‘yeah we’ll meet up with you’” (UK focus group 2)

Some Facebook groups and apps used had been purposefully created to facilitate meeting up. This interlinking of the virtual to the real world was felt to be extremely valuable:

“It’s [mummy meet up app] a really nice idea[…] Because sometimes mums are quite lonely. All their friends are working or don’t have children yet”. (UK interview 3)

However, many had reservations about meeting others they had met online:

“That’s what I’d be a bit concerned about, strangers and the security. Because they could be anyone”. (UK Focus group 2)

Despite reservations, there is potential for technology to facilitate social connections. It was felt that the groups and apps would be more successful in promoting real world relationships if they matched people on their child’s age, location, or even cultural background:

“I think that online support groups can be good, but I think it’s nicer if they’re within your radius. … These are other moms that had a baby the same day as you, the week after you, the week before you, there all here in [name of place]. Like these are people you might actually see in the grocery store. Because I think that when you’re a new mom and you’re really overwhelmed, you can feel this disconnect with online forums sometimes. So to make it sort of … a little more local, a little more building a baseline of something you have in common”. (US interview 4)

### Social anxiety

Some women discussed social anxiety as a barrier to meeting other mothers. The online virtual relationships in forums were easier to maintain through textual exchanges and online persona, compared to meeting up, which was a more pressurized interaction:

“I saw, when I was pregnant, in the waiting room there was an app that can like connect you with other mums and things. I think it’s quite a good idea, but I don’t think I’d do it, because I like my little group of people. I don’t like going out to strange places and meeting lots of other groups of people”. (UK Interview 2)

It was felt that the meet up apps or groups would be more attractive if they incorporated physical activity, such as a walking group. This might overcome the concerns around security and be less awkward, as this would give meeting a purpose:

*“That would be good because obviously you’re in a public place, you’re not going to meet someone at someone’s house potentially … I would be more comfortable meeting out and about than going to say someone’s house”*. (UK Interview 1)

### Integration

#### The “new norm”

The final phase of integration represented a move beyond acceptance, towards fully integrating the mothering identity within the self. Most women over time described how they came to see themselves as mothers. This major identity change was an integrated role that became one of the multiple identities that women simultaneously display and deploy. With regards to physical activity in particular, positive behaviours were often “just a part of life”. Integration of the mothering role involved simultaneous integration of health behaviours, adapting in individualized ways to the “new norm”. Although this was not always recognized as engagement in positive health behaviour (as it was not purposeful), the outcome was positive:

“I mean does going to work count? Or taking two babies up and down stairs and to parks and stuff, does that count? I haven’t done actual exercise, no.” (US Int 1)

For some women however, engaging with technology did not support acceptance or integration of health behaviours with a new, changed identity as a mother:

*“I do feel judged by my Fitbit every single day. I never hit 10,000 steps.” (US FG1)*Integration of the Mothering identity also involved a “new level” of organization and support needs, which were influenced by structural and environmental factors:

“Once you have to start pumping and bottle feeding because you’re away from your baby it becomes this whole new like level of, like, requiring organisation, and you know, attention and mental energy”. (US FG2)

Integration was also influenced by the infant’s behaviour and needs in relation to the Mother, and environmental factors, that on the one hand could be supportive, but on the other were stigmatizing and made it difficult to engage in breastfeeding, for example, as a positive health behaviour:

“I’ve also been to other places like [shop name] has a room which I thought was a wonderful concept but the room is an old changing room and there’s still the gap underneath and there’s people walking around outside and they have nothing on the wall, you’re just in a white cubby, which is super awkward because you just feel like you’re in like a little prison. I would’ve honestly been more comfortable walking around the store nursing my baby than sitting in that weird room. (US FG1)”

### Felt stigma

“Felt” stigma (Gray, 2002) and embarrassment at breastfeeding in public were reiterated across our UK and US sample. In integrating new health behaviours as part of the mothering identity, women discussed the use of technology in supporting lifestyle change:

“I know it sounds ridiculous but I’ve got an app on my phone. So I put my … the day that I was going to quit. I didn’t quite fully quit on that week, I then still had the odd cigarette and that at the end of the day was like ‘well done, you’ve won one day, you’ve saved this amount of money, you’ve saved like an hour’ like because obviously I don’t smoke indoors, like ‘you would’ve spent an hour outside smoking in your day’. And it’s kind of made me think ‘oh gosh yeah’, you know, I probably would’ve. You know, an hour out of my day would’ve been stood on the door step smoking. And it kind of wills you on and then it’s like … you get like an award like for the first day, won, and second day, won, and then so many hours which I thought is quite nice because they day you’re … you know sometimes when I was tempted I’d got on the app because it tells you exactly the hours … it does an award but that’d tell you like hours so that always looks more and I’m like ‘oh wow, you know I really want a cigarette’ but that’s like I’ve, you know, *I’ve done a hundred and fifty hours—do I really want to go back?” (UK FG 1)*

### Normalized use of technology

Engagement with technology is a normalized activity for postpartum women, and because of this, it was discussed as aiding the integration of healthy behaviours into the everyday mothering role, in this case through the accumulation of knowledge:

“I’m pretty much in my Facebook group all day. It’s a mommy group so we’re all science-based. So yeah, I mean, I do like … I do a lot of research, I read a lot of links on like breastfeeding or peaceful parenting or car seat safety”. (US Interview 2)

Technology offered opportunities to fit exercise into daily routines within the comfort of home, which was especially important given that lack of childcare was often cited as a barrier:

“I have my [name of programme] subscription through my phone as well. So I can access any workouts or track my progress.” (US interview 5)

Communication technology such as WhatsApp or Skype was discussed by both UK and US women as maintaining family communication, as well as keeping in touch with other mothers they knew from real world baby groups, who could offer additional virtual support and reassurance:

“I’ve got a few WhatsApp groups with some of the people I’ve met in like the baby classes we’ve been to.[…] Yeah like at the end … we did like the course through the [name of group] or whatever and they encouraged you to set up a WhatsApp group with the people that … as it was our last one because it was only a six week thing, they kind of said “why don’t you have a WhatsApp” so that was a way of kind of starting making some new friends.” (UK FG 2)

Tracking was another way postpartum women used technology to integrate healthy behaviours into their daily routines. Pedometers were widely used to monitor levels of physical activity. They also mentioned the usefulness of tracking breastfeeding to help feel less daunted by a task which could often feel relentless:

“When you’re doing it [breastfeeding] some days when you’re tired and you’re doing it … because some days they’re really hungry or they want comfort, you’re doing it constantly. Sometimes it’s quite hard to remember which side you fed … Yeah so I think that’s quite handy, to easily log it in.” (UK FG 1)

A few women discussed the potential usefulness of an app that might help them integrate multiple postpartum health behaviours into their daily lives simultaneously:

“I think if it’s a one-stop shop sort of thing that would be handy because then you haven’t got to download half a ton of apps for different things. If you can just go to one place, I think it would make life a lot easier. (UK interview 1)

Having technology to ease the burden of “information overload”, particularly at the point of integration of the mothering identity and beginning to feel at ease with being a mother, was seen as something that could be potentially extremely useful and supportive.

It was further suggested that technology might also help integrate behaviours into the motherhood role by supporting everyday “real world” interactions to promoting intersectional health behaviours. For example, women discussed the usefulness of an app which would use GPS to locate the nearest breastfeeding room, child-friendly café, or playground. This app could help support mothers to breastfeed during physical activity such as walking with the baby, without the worry of being judged or having a hungry baby and not knowing where they could feed them:

“That would’ve been brilliant, especially when I had my first, because I was in our local mall trying to breastfeed and someone got quite narky with me and said “oh you shouldn’t have them out” … If there was a list [of public places for breastfeeding] that would be brilliant. If that was in an app that would be brilliant. I think that would empower a lot more mums to potentially want to breastfeed if they knew of places where they could go and breastfeed”. (UK Interview 1)

Apps could also promote physical activity by encouraging mothers to leave the house and explore their local areas:

“Some parks there isn’t really much going on and [name of child] likes the swings so like if you could see that there’s swings at this park, then yeah definitely I’d like try different places and go further afield as well. That would be quite good.” (UK Interview 2)

Women also felt that technology could be used to bring organized weekly parent groups together and integrate them into their routines.

“I think if it was like a set time and happened each week that would be quite good. I think if it was random I don’t think people would really say ‘oh I’m just going to … ’ because like people have to plan things and … but if it was like structured and each week I think that would be quite good to then do something. Because then you get to know people.”. (UK Interview 2)

Technology also supported social meet up groups for US women, although it was felt that these were often targeted towards non-working mothers:

“As a working mother I feel like I’m always trying to get people on board to go out and walk or do activities or look in the paper, see what’s going on locally, and everything’s at like 10am.” (US FG 2)

## Discussion

Our findings demonstrate the important underlying concept of intersectionality (Bauer, 2014)—a theoretical construct suggesting that lifestyle behaviours that represent cancer risk behaviours are complex, interrelated, and interact in multiple ways—to impact cancer preventative health behaviours following childbirth across US and UK women. The postpartum period is a life-stage process of identity in transition, moving through key transitional moments: disturbance to the known identity, adaptation, acceptance and integration. At each stage women experienced multiple barriers to engaging in healthy cancer preventative behaviours, interacting with each other. In our UK dataset, women particularly discussed a lack of support to stay smoke-free, corresponding to review findings that women need social support, especially from a partner, to stay smoke-free (Notley et al., 2015). The difficulty of finding time to engage in physical activity, and the need for access to physical spaces for exercise, was particularly discussed in our US dataset. Across both datasets, women discussed the challenges of learning to breastfeed, supporting review evidence of the need for evidence-based support interventions for continued breastfeeding (Kassianos et al., 2019) Continued breastfeeding, particularly supporting “pumping” breastmilk, seemed to be more supported in the US than in the UK context, as might be expected reviewing the low UK breastfeeding rates (*Breastfeeding in the UK, UNICEF, 2019*).

Consistent with existing research, both US and UK women were regular and skilled users of technology, using apps to track fitness (steps), accessing information on breastfeeding via websites and apps, and using electronic means of forming and maintaining social networks, and linking online to “real world” social groups (Johnson, 2015; Kurti et al., 2019). In general, the qualitative data revealed that technology was a facilitator—a means to promote positive health behaviours and to provide support for women adapting to a postpartum identity. However, there were cases in which technology was a barrier, for example, through women’s engagement with negative peer interactions using social media, or through the proliferation of knowledge that women had trouble discriminating as “evidence-based” or “trustworthy” as opposed to “folk-knowledge”.

Our results clearly demonstrate interactions of cancer preventative behaviours in the postpartum period that have been well documented in previous studies. Women discussed breastfeeding as a barrier to physical activity (Evenson et al., 2009). They also discussed wanting to stay smoke-free, recognizing this as positive health behaviour change, but struggled with stress and lack of social support, both of which predict postpartum smoking relapse (Orton et al., 2017). The ability for women to enact interrelated cancer preventative behaviours was influenced by factors at all levels of the social ecological model, although key influencers occurred at the interpersonal (small group or family) level, and to a lesser extent also at the individual (motivational) level. US and UK women were more similar than different, although some differences particularly in breastfeeding support and physical activity limitations were noted. This suggests that interventions to support cancer preventative health behaviours postpartum need to equally emphasize social alongside individual support, combined with societal and cultural influence, for example, through consideration of the local built environment where women can integrate healthy behaviours into their daily routines.

Women discussed the isolation of adapting to the new mothering role before integration was achieved. Technology had a clear role, for virtual support, and to facilitate “real world” support. This type of generic social support was accessible to the women in our predominantly low SES sample, who may otherwise have had limited opportunities for engagement, and likely experienced multiple inequalities, such as lack of access to healthcare and education. Generic social support can be understood as having a positive but complex and intersectional impact on the enactment of healthy behaviour postpartum. Bolstering self-esteem, self-efficacy and a general feeling of support are all critical to engagement in positive health behaviours including increased physical activity, confidence in ability to breastfeed, and confidence in ability to remain smoke-free.

This exploratory qualitative study was limited by the convenience sample, despite attempts to purposively sample and ensure that a variety of viewpoints were captured. Due to the nature of the sample and project aims, the findings cannot be considered generalizable, but are nonetheless transferable and informative for intervention development. The process of analysis was thorough and rigorous, with double coding of all data (Barbour, 2001).

Technologies designed to directly support breastfeeding, smoking abstinence or physical activity were primarily experienced in isolation, as, for example, targeted apps for each behaviour. However, given the complex interactions between cancer preventative behaviours postpartum, there is a clear need to develop an intervention to support health behaviours in combination, and in a situated, meaningful way that makes sense to women in their unique contexts. Particularly, interaction between technology and “real world” support, i.e., linking women through virtual social support groups to actual support groups in their local environments, or offering support to continue breastfeeding through social media or online support groups, and pinpointing breastfeeding friendly venues in women’s immediate geographical areas. There is great potential for technology to support positive health behaviour change cross-culturally, through recognizing interrelationships and providing innovative digital/electronic social support and motivational tools that ultimately can impact on cancer prevention outcomes for both women and infants.

## Conclusions

The postpartum period is a time of intense identity transition. For UK and US Mothers, this combines with pressures and expectations about engaging in health behaviours that support positive health outcomes for the mother and infant. Whilst technology was accessed and well used by women in our study to support positive health behaviours, these were primarily accessed as isolated support focused on one health behaviour, or more generically, as a means of postpartum social support. However, the lives of Mothers are complex and relational—health behaviours interact along the process of identity change, and are supported or hindered through relationships with others. New technologies show great promise and potential to support the complex, multiple and intersectional needs of postpartum women, but need to be responsive, adaptive, tailored and multi-functional—responding to individual need whilst supporting women within the contexts of their unique social and cultural worlds.

## Supplementary Material

Supplemental Material

## Figures and Tables

**Figure 1.**
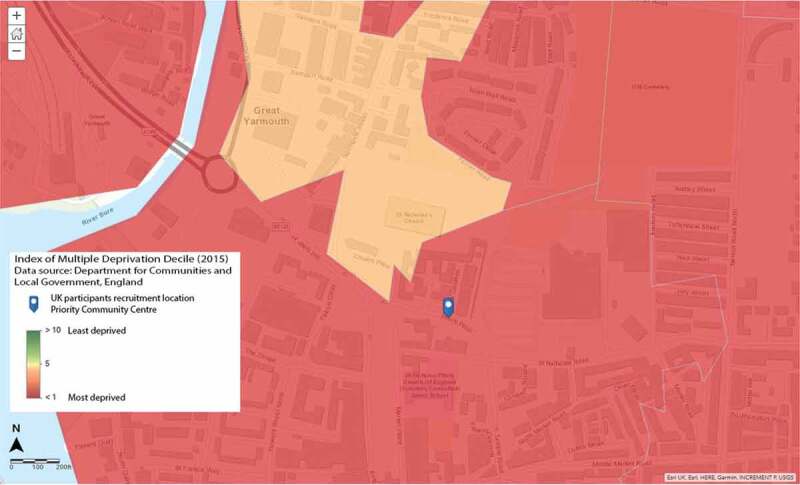
UK participants recruitment location 2.

**Figure 2.**
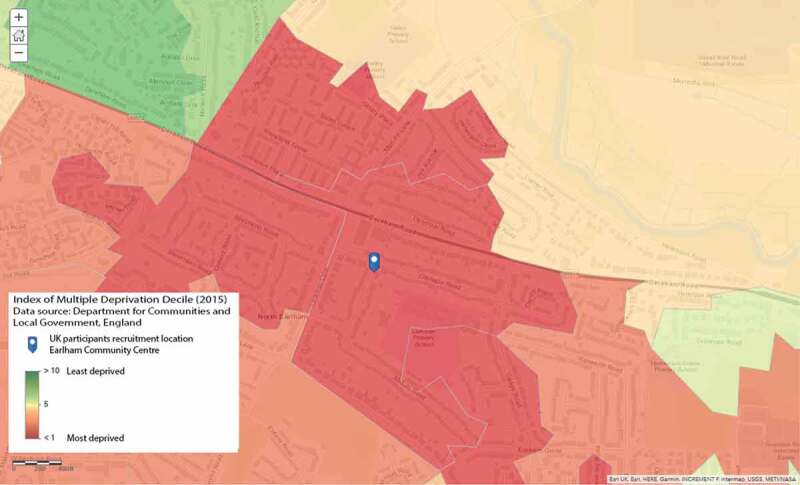
UK participants recruitment location 2.

**Figure 3.**
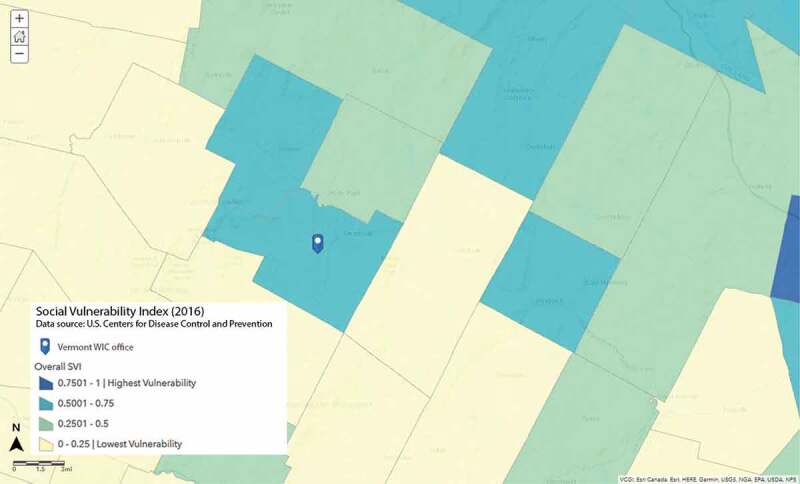
US participants recruitment location 1.

## Appendix A – Supplementary recruitment information

No home addresses were collected, for privacy purposes. Participants were recruited at service providers in both countries. To provide demographic context, we use indices to measure social vulnerability relevant to each country.

UK participants were recruited at two Children’s Centres (Figures 1 and 2). Children’s Centres are Government funded organisations offering early years’ education and support for parents of infants and young children, with the aim of improving child outcomes and reducing inequalities. Children’s Centres have catchment areas and parents are usually registered with the Children’s Centre located closest to their home address.

The Department for Communities and Local Government developed the Index of Multiple Deprivation (IMD) based on seven categories: income, employment, education, skills and training, health and disability, crime, barriers to housing and services, and living environment. The IMD ranks every small area in England most often showing where an area falls in the ‘deciles’ compared to the national average, with 1 as the most deprived area. Both of the recruitment locations are within the top IMD deciles, meaning that they fall among the most deprived 10 percent of small areas in England.

US participants were recruited through an affiliate Women, Infants, and Children (WIC) office. The US federal government funds the WIC program to provide supplemental foods, health care referrals and nutrition for low-income women beginning from pregnancy to children up to age five. WIC offices are often strategically located in areas that can best serve low-income families. Figure 3 shows how the census tract of the location where most participants were recruited ranked in the Social Vulnerability Index (SVI). The SVI is based on U.S. Census data that ranks each census tract into four general categories of socioeconomic, housing composition and disability, minority status and language, and housing and transportation. Our participants were recruited in a location with moderate to higher level vulnerability across all categories.

US participants were recruited through an affiliate Women, Infants, and Children (WIC) office. The US federal government funds the WIC program to provide supplemental foods, health care referrals and nutrition for low-income women beginning from pregnancy to children up to age five. WIC offices are often strategically located in areas that can best serve low-income families. Figure 3 shows how the census tract of the location where most participants were recruited ranked in the Social Vulnerability Index (SVI). The SVI is based on U.S. Census data that ranks each census tract into four general categories of socioeconomic, housing composition and disability, minority status and language, and housing and transportation. Our participants were recruited in a location with moderate to higher level vulnerability across all categories.
