# Supplementary material for: Negotiating cancer preventative health behaviours and adapting to motherhood: the role of technology in supporting positive health behaviours
Source: Int J Qual Stud Health Well-being. 2020 Sep 18;15(1):1811533. doi: 10.1080/17482631.2020.1811533 (PMC7534276; doi:10.1080/17482631.2020.1811533)
Supplement: Supplemental Material [file ZQHW_A_1811533_SM8356.docx]

**Appendix A – Supplementary recruitment information**

No home addresses were collected, for privacy purposes. Participants were recruited at service providers in both countries. To provide demographic context, we use indices to measure social vulnerability relevant to each country.

UK participants were recruited at two Children’s Centres (Figures 1 and 2). Children’s Centres are Government funded organisations offering early years’ education and support for parents of infants and young children, with the aim of improving child outcomes and reducing inequalities. Children’s Centres have catchment areas and parents are usually registered with the Children’s Centre located closest to their home address.

The Department for Communities and Local Government developed the Index of Multiple Deprivation (IMD) based on seven categories: income, employment, education, skills and training, health and disability, crime, barriers to housing and services, and living environment. The IMD ranks every small area in England most often showing where an area falls in the ‘deciles’ compared to the national average, with 1 as the most deprived area. Both of the recruitment locations are within the top IMD deciles, meaning that they fall among the most deprived 10 percent of small areas in England.

| 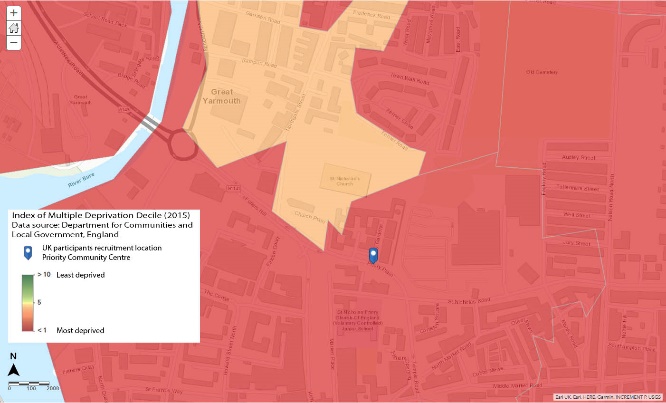  Figure 1 UK participants recruitment location 2 | 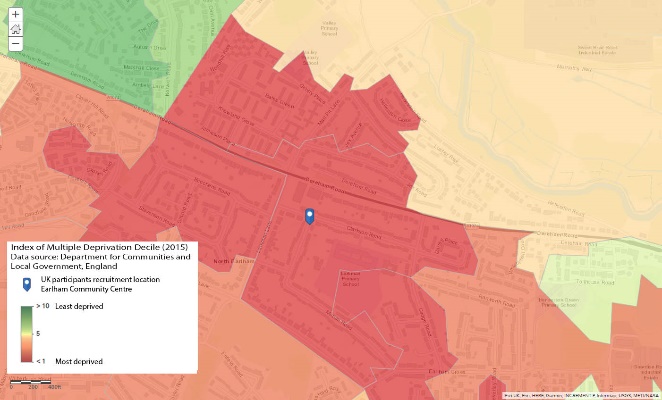  Figure 2 UK participants recruitment location 2 |
| --- | --- |

US participants were recruited through an affiliate Women, Infants, and Children (WIC) office. The US federal government funds the WIC program to provide supplemental foods, health care referrals and nutrition for low-income women beginning from pregnancy to children up to age five. WIC offices are often strategically located in areas that can best serve low-income families. Figure 3 shows how the census tract of the location where most participants were recruited ranked in the Social Vulnerability Index (SVI). The SVI is based on U.S. Census data that ranks each census tract into four general categories of socioeconomic, housing composition and disability, minority status and language, and housing and transportation. Our participants were recruited in a location with moderate to higher level vulnerability across all categories.


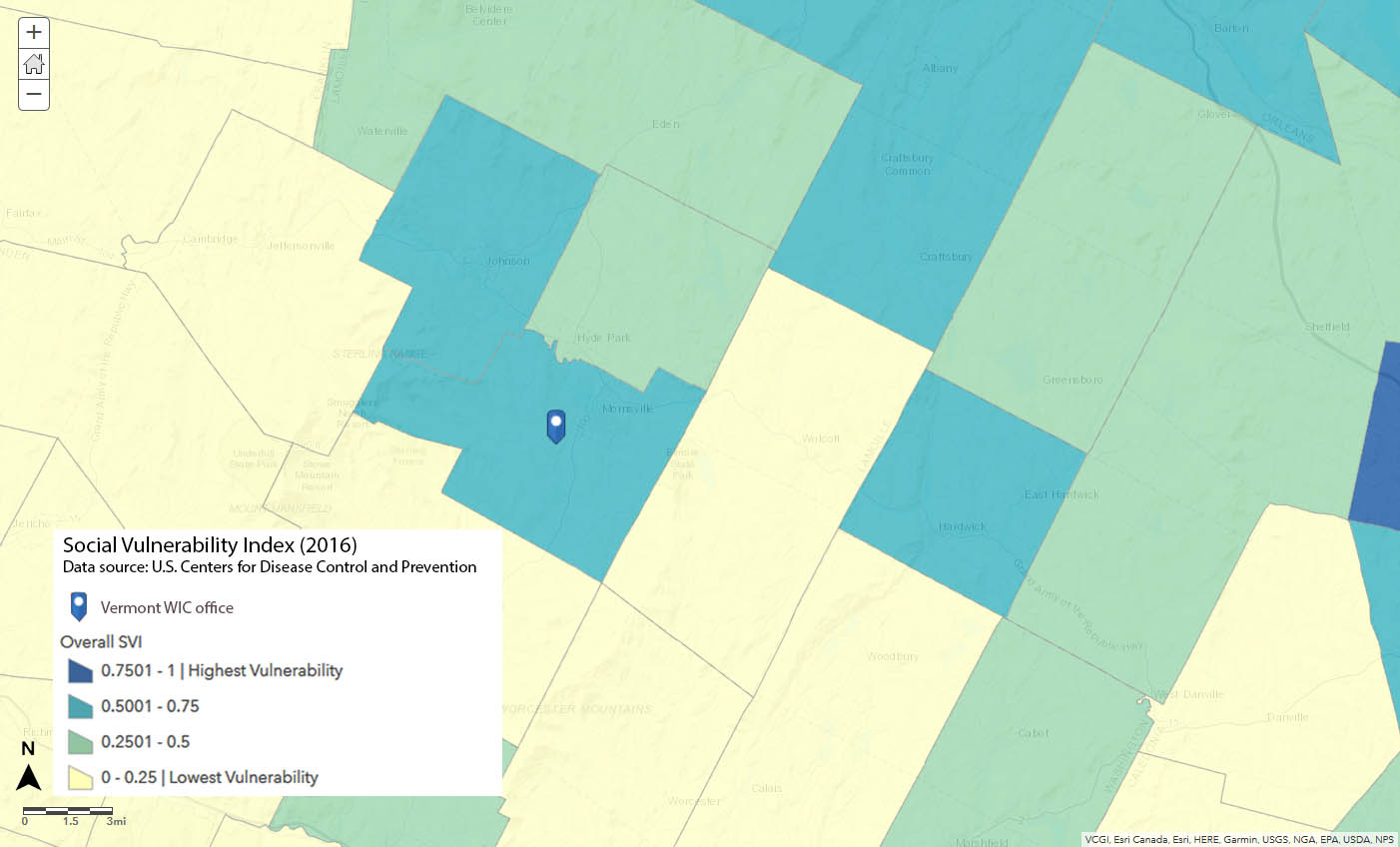


Figure 3 US participants recruitment location 1
